# The novel mu-opioid antagonist, GSK1521498, reduces ethanol consumption in C57BL/6J mice

**DOI:** 10.1007/s00213-015-3995-x

**Published:** 2015-07-05

**Authors:** Tamzin L. Ripley, Sandra Sanchez-Roige, Edward T. Bullmore, Manolo Mugnaini, Kay Maltby, Sam R. Miller, David R. Wille, Pradeep Nathan, David N. Stephens

**Affiliations:** School of Psychology, University of Sussex, Falmer, Brighton BN1 9QG UK; Department of Psychiatry, University of Cambridge, Addenbrooke’s Hospital, Cambridge, UK; Academic DPU, Alternative Discovery and Development, GlaxoSmithKline R&D, Addenbrooke’s Hospital, Cambridge, UK; Quantitative Sciences, GlaxoSmithKline R&D, Stevenage, UK; Translational Pharmacology, Aptuit, Verona, Italy; Biology Department, Neuroscience Discovery, AbbVie Deutschland GmbH & Co. KG, Ludwigshafen, Germany

**Keywords:** Naltrexone, Drinking-in-the-dark, Receptor occupancy, Inverse agonist, Mu-opioid receptor

## Abstract

**Rationale:**

Using the drinking-in-the-dark (DID) model, we compared the effects of a novel mu-opioid receptor antagonist, GSK1521498, with naltrexone, a licensed treatment of alcohol dependence, on ethanol consumption in mice.

**Objective:**

We test the ability of GSK1521498 to reduce alcohol consumption and compare its intrinsic efficacy to that of naltrexone by comparing the two drugs at doses matched for equivalent receptor occupancy.

**Methods:**

Thirty-six C57BL/6J mice were tested in a DID procedure. In 2-day cycles, animals experienced one baseline, injection-free session, and one test session when they received two injections, one of test drug and one placebo. All animals received GSK1521498 (0, 0.1, 1 and 3 mg/kg, i.p., 30 min pre-treatment) and naltrexone (0, 0.1, 1 and 3 mg/kg, s.c. 10 min pre-treatment) in a cross-over design. Receptor occupancies following the same doses were determined ex vivo in separate groups by autoradiography, using [3H]DAMGO. Binding in the region of interest was measured integrally by computer-assisted microdensitometry and corrected for non-specific binding.

**Results:**

Both GSK1521498 and naltrexone dose-dependently decreased ethanol consumption. When drug doses were matched for 70–75 % receptor occupancy, GSK1521498 3 mg/kg, i.p., caused a 2.5-fold greater reduction in alcohol consumption than naltrexone 0.1 mg/kg, s.c. Both GSK1521498 and naltrexone significantly reduced sucrose consumption at a dose of 1 mg/kg but not 0.1 mg/kg. In a test of conditioned taste aversion, GSK1521498 (3 mg/kg) reduced sucrose consumption 24 h following exposure to a conditioning injection.

**Conclusions:**

Both opioid receptor antagonists reduced alcohol consumption but GK1521498 has higher intrinsic efficacy than naltrexone.

**Electronic supplementary material:**

The online version of this article (doi:10.1007/s00213-015-3995-x) contains supplementary material, which is available to authorized users.

## Introduction

Alcoholism is a complex heterogeneous disorder, for which there are currently limited treatment options. While it is routine for alcohol dependent patients to undergo clinically supervised detoxifications, maintaining abstinence is frequently unsuccessful, leading to successive repeated episodes of further detoxification and relapse, with ever-increasing difficulty in maintaining abstinence (Loeber et al. 2010). One of the few strategies currently licensed for reducing relapse is the blockade of mu-opioid receptors (Kiefer et al. 2003; Volpicelli et al. 1992). Although there is good evidence that naltrexone significantly reduces the risk of drinking (Monti et al. 1999), the overall effectiveness is limited, with risk of relapse showing only a moderate decline from an untreated relapse risk of 1.00 to a relative risk of 0.83 (Rosner et al. 2010). In the majority of studies published, naltrexone reduces significantly the rates of drinking in heavy drinkers by 30–60 %. While naltrexone reduces craving significantly, abstinence is achieved usually in only 25–35 % of cases (Pettinati et al. 2006). The most recent meta-analysis (Donoghue et al. 2015) found that the risk of individuals returning to any drinking at approximately 3 months was significantly reduced for the naltrexone group (RR = 0.92, 95 % CI = 0.86–1.00) as was the risk of individuals relapsing to heavy drinking at 3 months (RR = 0.85, 95 % CI = 0.78–0.93).

Nevertheless, the therapeutic utility of naltrexone in alcohol dependence is limited, perhaps as a result of clinical and genetic heterogeneity: e.g. effects of naltrexone are stronger in the subgroup of alcoholic patients carrying the Asp variant of the mu-opioid receptor (OPRM1) A1118G SNP (Oslin et al. 2003). It may also be relevant that naltrexone is rapidly metabolised to 6-β-naltrexol and that both these drugs have partial agonist activity at mu-opioid receptors under some assay conditions (Kelly et al. 2014). It is also notable that adherence to naltrexone treatment is poor, and this is often attributable to adverse events including nausea and vomiting (de Wit et al. 1999).

GSK1521498 is a novel mu-opioid receptor antagonist that is being developed for disorders of compulsive reward-driven behaviour. Using recombinant human opioid receptors, we have previously observed in vitro that GSK1521498 is a selective mu-opioid receptor antagonist with approximately 14-fold selectivity for mu over both delta- and kappa-opioid receptor subtypes (Ignar et al 2011; Kelly et al 2014). Compared to naltrexone, GSK1521498 was more selective for the mu-opioid receptor and had greater affinity for mu-opioid receptor binding. We also observed that whereas GSK1521498 could completely antagonize mu-opioid receptor activation by an exogenous agonist challenge, naltrexone, naloxone, and 6-β-naltrexol could achieve only about 70 % blockade even at high doses, and whereas GSK1521498 had slight inverse agonist activity, naltrexone had partial agonist activity, in cell lines overexpressing mu-opioid receptors (Ignar et al. 2011; Kelly et al. 2014). The human pharmacokinetics and brain receptor occupancy of GSK1521498 are not so directly relevant to this study but have been reported elsewhere in comparison to naltrexone and 6-β-naltrexol (Nathan et al. 2012a, b; Rabiner et al. 2011). The behavioural effects of GSK1521498 have been reported using second-order reinforcement schedules to elicit drug-seeking behaviour in rats trained to work for self-administration of heroin or cocaine (Giuliano et al. 2013). At equivalent doses to those used in the current study, GSK1521498 demonstrated significantly greater efficacy than naltrexone in inhibiting drug-seeking behaviour for cocaine and heroin rewards.

Here, we aimed to test the intrinsic efficacy of GSK1521498 for the first time in an animal model of alcohol consumption, and, specifically, to compare the efficacy of GSK1521498 to that of naltrexone with the two drugs matched both for dose and for receptor occupancy. The drinking-in-the dark (DID) paradigm has been shown to be an effective way to induce alcohol consumption (Hwa et al. 2011; Ripley and Stephens 2011). In this model, C57BL/6J mice, which are genetically predisposed to drink ethanol, are given access to 20 % ethanol solution, replacing their water bottles, for 2 h beginning 3 h after the start of their dark phase. In the majority of animals, this leads to an ethanol intake that produces high blood ethanol levels and signs of behavioural intoxication (Rhodes et al. 2007). This model has been evaluated using drugs that have been shown to reduce ethanol consumption in other animal models and in humans. Naltrexone has been shown to be efficacious in this model (Kamdar et al. 2007) with doses of 0.5, 1, or 2 mg/kg dose-dependently decreasing ethanol consumption without significant effect on the consumption of plain water or 10 % sugar water. On the basis of the prior pharmacological and behavioural data on GSK1521498, we predicted hypothetically that it would also reduce alcohol consumption in this model and that it would have higher intrinsic efficacy than naltrexone.

In the first experiment, we compared the effects of GSK1521498 and naltrexone on ethanol consumption in ethanol-experienced C57BL/6J male mice. To control for non-specific effects of both compounds, we also examined the effects of GSK1521498 and naltrexone on consumption of a sucrose solution in the same experiment. Decreases in consumption of ethanol could be due to non-specific effects, such as general malaise following the injection, and there is some evidence to suggest that naltrexone may also produce a conditioned taste aversion (CTA) at relatively low doses (Parker and Rennie 1992). In a second experiment, we therefore tested GSK1521498 in a CTA paradigm and compared it against a standard dose of lithium chloride. Finally, in order to evaluate possible differences in intrinsic efficacy of GSK1521498 and naltrexone with the drugs matched for receptor occupancy, we additionally quantified binding of GSK1521498 and naltrexone binding to mu-opioid receptors (MOPr) in mouse brain using autoradiography. We reasoned that demonstrating a superior behavioural effect of GSK1521498 versus naltrexone at equivalent levels of receptor occupancy would provide more rigorous evidence of greater intrinsic efficacy of GSK1521498 than a comparison matched in terms of administered doses (Jones et al. 1994).

## Materials and methods

### Animals

In all studies, C57BL/6J male mice (Charles River, Margate, UK), weighing approximately 20 g at the start of the experiment, were singly housed in standard caging containing sawdust and corrugated paper bedding material. Mice had ad libitum access to food (Purina lab chow) and water throughout the experiment unless otherwise stated. The facility was maintained at a temperature of 21 ± 2 °C and humidity of 50 ± 5 %. Animals were maintained on a 12-h light/dark cycle with lights on at 7 am, unless otherwise stated. All experiments were conducted in accordance with the UK (1986) Animal (Scientific Procedures) Act (Project Licence 70/7072) and the GSK Policy on the Care, Welfare and Treatment of Laboratory Animals.

The numbers of animals used in each study were as follows: DID study in ethanol experienced animals, *N* = 36; conditioned taste aversion study, *N* = 32; and receptor occupancy study, *N* = 40.

In the DID experiment, nine animals did not complete the study. Of these animals, two were unwell at the time of purchase and were removed from the study during the home cage drinking phase. The remaining animals were removed during the DID phase of the experiment due to ill health, although this attrition was not evidently related to drug treatment.

### Home cage ethanol consumption using the intermittent ethanol escalation model

Home cage access to ethanol followed the intermittent schedule described by Hwa et al. (2011). In brief, mice were given intermittent access to ethanol for 24-h periods in a two-bottle choice paradigm. Access to one bottle containing ethanol and one containing water was on Monday, Wednesday and Friday. After 24 h, the bottles were removed and weighed and replaced with two bottles of water. The two water bottles remained in place until the next ethanol drinking session. The placement of bottles was alternated before each ethanol drinking session to avoid side preferences. Over the first four experimental sessions, ethanol concentration was increased in each successive session (3, 6, 10 and 20 % (*v*/*v*)). The concentration was then maintained at 20 % for 3 weeks.

Mice were divided into two groups (group A (*n* = 12) and group B (*n* = 15)) based on ethanol consumption during the home cage ethanol escalation phase. Groups were balanced so that there was no significant difference in alcohol consumption between the two groups. These groups determined the order in which the animals received drug treatment during the test phase of the experiment.

### Effect of GSK1521498 and naltrexone on ethanol consumption using the DID procedure

**Fig. 1:**
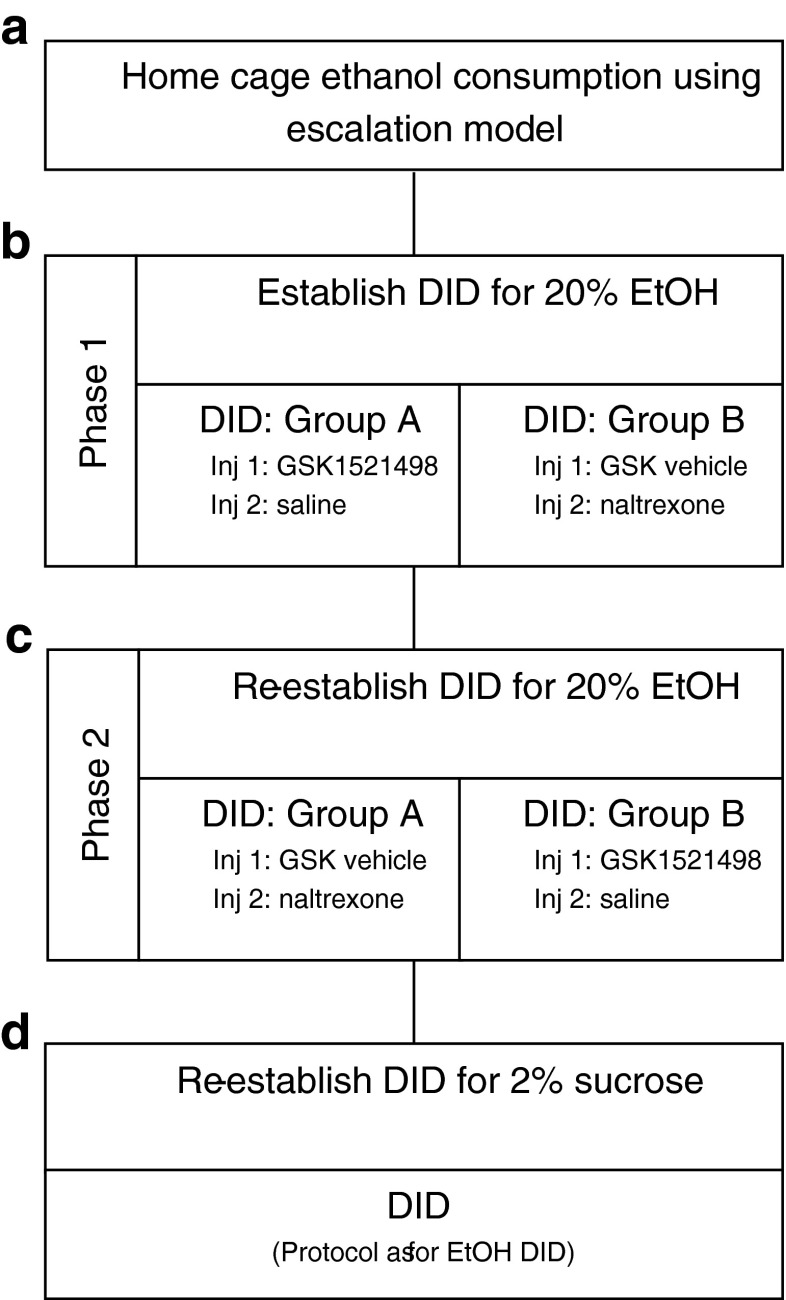
Overview of the experimental protocol. The experiment was divided into four parts: **a** animals consumed ethanol in their home cage using the ethanol escalation model; **b** phase 1: following the establishment of baseline ethanol consumption using the DID method animals were divided into two groups with group A receiving GSK1521498 and group B receiving naltrexone as the test compound; **c** phase 2: following a 2-week wash-out period, DID was re-established and a cross-over design was employed such that group A received naltrexone and group B received GSK1521498 as the test compound; **d** compound specificity was tested using a 2 % sucrose DID protocol

Following home cage ethanol consumption using the intermittent ethanol escalation model, and 1-week habituation to the shifted light/dark cycle (lights off at 11 am), the animals were trained to drink in the dark a 20 % ethanol solution from spring loaded sipper tubes over a 3-week period, using a 2-day intermittent DID procedure (Kamdar et al. 2007; see Fig. 1 for an overview of the DID protocol).

In brief, mice were exposed to the DID procedure on 4 days each week. Starting 2 h after lights off, water bottles were removed and the cages placed on the experimental table. The animals were left undisturbed for 1 h and then a spring-loaded sipper tube attached to a 10-ml graduated pipette containing the experimental solution was inserted into the nozzle space in the cage lid, where the water bottle was usually placed. Animals were left undisturbed for a further 2 h. After this period, the volume of solution consumed was measured by reading from the bottom of the liquid meniscus in the pipette. Cages were returned to the holding rack and water bottles were replaced.

Following the establishment of baseline ethanol DID consumption (Fig. 1a), the animals were divided into two groups: group A, receiving GSK1521498 (0, 0.1, 1 and 3 mg/kg; i.p., 30 min pre-treatment) and saline (s.c., 10 min pre-treatment); group B, receiving GSK1521498-vehicle (i.p., 30 min pre-treatment) and naltrexone (0, 0.1, 1 and 3 mg/kg; s.c., 10 min pre-treatment). In order to habituate the mice to the injection protocol, prior to drug testing, mice were exposed to three sessions where they received two injections of saline (i.p. 30 min; s.c. 10 min prior to drinking; Tuesday, Friday, Tuesday), followed by three additional injection sessions where they received GSK1521498 vehicle (30 min i.p.; naltrexone vehicle, 10 min prior to drinking, s.c.; Friday, Tuesday, Friday).

Next, over a 4-week period (phase 1; Fig. 1b), mice experienced two weekly baseline sessions, and two weekly test sessions where they received two injections (GSK1521498 and saline, or GSK1521498-vehicle and naltrexone; Tuesday, Friday). Analysis was carried out on the average consumption across the two baseline and two injection sessions for each week.

At the end of the 4 weeks of drug treatment, animals were given a 3-week ethanol/injection-free period, to allow for drug wash out. Subsequently, animals were then allocated to the opposite drug group and DID behaviour was established over a further 4-week period (phase 2; Fig. 1c). The protocol followed the procedure described above except that for the first 2 weeks, animals were not injected, and for the second 2 weeks, animals received two injections (GSK1521498-vehicle, i.p., 30 min pre-treatment; saline, s.c. 10 min pre-treatment) on Tuesday and Friday.

### Effect of GSK1521498 and naltrexone on sucrose consumption using the DID procedure

Animals were given a further 3-week ethanol/injection-free period before being tested to see if naltrexone or GSK1521498 altered sucrose consumption (Fig. 1d). Using the DID paradigm, animals had access to 2 % sucrose solution in the sipper tubes. Animals were habituated to the procedure for four sessions, without injection. The procedure then followed stages as described above with baseline (non-injection days) on Monday and Thursday and injection days on Tuesday and Friday. On these days, the animals received the two-injection protocol previously described. The doses tested were 0, 0.1 and 1 mg/kg for both GSK1521498 and naltrexone. The doses were administered in a pseudo-random order with all mice receiving all doses. Each dose was only tested for one session.

### Effect of GSK1521498 and naltrexone on the development of conditioned taste aversion to sucrose

Naive animals were divided into four groups, each of which received either lithium chloride (256 mg/kg, 0.6 M, i.p.) or a dose of GSK1521498 (vehicle, 1 or 3 mg/kg, i.p.).

The CTA experiment followed standard protocols previously used in our laboratory (Stephens and Dunworth 2000). For 7 days, animals were water restricted to 4-h access to water in their home cages (between 10 am and 2 pm). This trained the animals to drink a large quantity of water during this time period.

On days 1 to 3, animals were habituated to the spring-loaded sipper tubes. Cages were removed from the holding rack at 10 am and placed on the experimental table. Spring-loaded sipper tubes attached to 10-ml graduated pipettes were inserted into the nozzle space in the cage lid, where the water bottle was usually placed. Animals were allowed to drink from the sipper tube for 30 min. The amount consumed was recorded by reading from the bottom of the liquid meniscus in the pipette. Cages were then returned to the holding rack, but animals were not given access to their water bottle for a further 1 h. Animals then had access to water for 3.5 h (between 11.30 am and 3 pm). Animals were allocated to test group based on the average amount that they had consumed over days 2 and 3 of this baseline period so that baseline consumption was matched across treatment groups.

On day 4, the conditioning day, cages were placed on the experimental table and sipper tubes, containing 10 % sucrose solution, were inserted into the cage for 30 min. Fluid consumption was measured. Animals were then immediately injected with either LiCl or GSK1521498 (as described above). Animals were returned to their home cage for 1 h before access to water bottles for 3.5 h (as on days 1 and 2).

Day 5, the test day, mimicked day 4 with the exception that animals did not receive injections. Day 6 was used to test the specificity of the conditioning for sucrose. The procedure mimicked day 5, except that water replaced sucrose in the sipper tubes.

### Receptor occupancy

Receptor occupancy was analysed using the protocol previously described by Codd et al. (2010). Animals were divided into two groups. The first group were treated with GSK1521498 (0, 0.1, 1, 3 or 10 mg/kg, i.p. (four mice per treatment group)) and killed by decapitation 1 h later. The second group were treated with naltrexone (0, 0.1, 1, 3 or 10 mg/kg, s.c. (four mice per group) and killed by decapitation, following cervical dislocation, 40 min later. These timings were chosen to match those in the drinking in the dark experiment.

On decapitation, the brains were removed, immediately frozen in pre-cooled 2-methylbutane (temperature −30 to −40 °C), and stored at −80 °C for up to 7 days. Brain slices (14-μm coronal sections) were cut at approximately +1.54 mm from bregma (Paxinos and Franklin 2001). Adjacent slices were mounted on glass slides and incubated at room temperature for 10 min with 5 nM [3H]DAMGO (specific activity 1850 GBq/mmol; PerkinElmer, UK) in buffer (50 mM Tris/HCl, 5 mM MgCl_2_, 0.1 % *w*/*v* bovine serum albumin, pH 7.4) containing 40 μg/ml autoclaved bacitracin, washed twice (at 4 °C) in buffer for 5 min, followed by deionised water (2 × 5 s), air dried and exposed to BAS-TR2025 imaging plates (Fuji Photo Film Co., Japan) for 3 weeks. Autoradiograms were generated using the Bio-image Analyzer BAS5000 (Fuji Photo Film Co., Japan), and the region of interest measured integrally by computer-assisted microdensitometry (MCID basic, Imaging Research, Canada). Photostimulated luminescence (PLS) per mm^2^ values were converted to the corresponding [^3^H]DAMGO concentration, expressed as fmol/mg brain tissue by reference to [^3^H] standards (Microscales, Amersham) on the same imaging plate. Of four slides for each brain, two were used for determination of total binding and two for non-specific binding (NSB), in the presence of 1 μM DAMGO, allowing specific binding to be calculated by subtraction. Percentage receptor occupancy (RO) of each GSK1521498- and naltrexone-treated mouse was calculated as RO(%) = [1 − SB_T_/SB_V_] × 100, where SB_T_ is the specific binding in each individual animal treated with drug, and SB_V_ is the mean SB for animals treated with vehicle. Occupancy data were calculated by non-linear regression analysis using GraphPad Prism V5.0., whereby RO(%) = [RO_max_ × (D)^γ]^/[(ROD_50_)^γ^ + (D)^γ^]., where D is the dose, ROD_50_ is the dose giving 50 % maximal occupancy (ROD_max_), and γ is the Hill coefficient of this function.

### Drug preparation

A stock solution of GSK1521498 (4 mg/ml, expressed in terms of the free base) in an acidified hydroxypropyl beta-cyclodextrin (HPBCD)-containing vehicle was provided by GlaxoSmithKline. All pre-prepared solutions were kept frozen throughout the duration of the experiment.

Stock GSK1521498 and vehicle blank solutions were thawed and diluted in the phosphate-buffered diluent to yield a 1 mg/ml solution. For the phosphate buffer, 4 g sodium chloride, 0.1 g potassium chloride, 0.44 g monobasic potassium phosphate and 0.241 g dibasic sodium phosphate (anhydrous) were diluted in 500 ml distilled water and mixed well.

This 1 mg/ml solution of GSK1521498 was diluted to produce solutions of 0.3, 0.1 and 0.01 mg/ml, using a serial dilution technique. Diluted solutions were filtered through a 0.2-micron filter prior to administration to the mice. Injection volumes were 10 ml/kg throughout. Final injection concentrations for GSK1521498 were vehicle, 0.1, 1 or 3 mg/kg, i.p., 30 min pre-treatment. Solutions were refrigerated and used within 48 h of preparation.

Naltrexone hydrochloride (Sigma-Aldrich, UK) was dissolved in saline to produce a 1 mg/ml solution. This 1 mg/ml solution was diluted to produce solutions of 0.3 and 0.1 mg/ml, using a serial dilution technique. Diluted solutions were filtered through a 0.2-micron filter prior to administration to the mice. Injection volumes were 10 ml/kg throughout. Final injection concentrations for naltrexone were vehicle, 0.1, 1 or 3 mg/kg, s.c., 10 min pre-treatment. Solutions were refrigerated after preparation and were used within 1 week of preparation.

Lithium chloride (LiCl: Sigma-Aldrich) was dissolved in saline to give a final concentration of 25.6 mg/ml. Injection volumes were 10 ml/kg throughout. The final injection dose for LiCl was 256 mg/kg, i.p. Solutions were refrigerated and used within 24 h of preparation.

### Statistical analysis

SPSS and SAS software were used for data analysis. Data transformations were applied for repeated measures ANOVA as described below to best meet the required assumption of sphericity. Residuals were observed to have magnitude approximately proportional to the mean. In these circumstances, it is appropriate to log-transform the data prior to analysis to normalise the size of the residuals. Not transforming would give more influence to high-value outliers and decrease precision around means at low drinking levels. Significant factorial effects from repeated measures ANOVA were explored using post hoc *t* tests.

Home cage ethanol drinking data were converted from millilitres to g EtOH/kg body weight for analysis. Consumption across the 12 home cage intermittent access drinking sessions was analysed using a repeated measures ANOVA with factors for animal group (A or B; between-subject) and drinking session (within-subject).

For the DID experiments, drinking data were converted from millilitres to g EtOH/kg body weight and log-transformed for analysis. Data were combined across phases 1 and 2 and were analysed using a repeated measures ANOVA with factors for drug, dose, phase and session (non-injection baseline vs test) and two- and three-way interactions (all within-subject). Magnitudes of effect were estimated with 95 % confidence intervals (quantified as fold changes following back-transformation of the log-transformation).

In order to make valid comparisons between the two test drugs, ethanol consumption data were further analysed taking into account receptor occupancy estimated from receptor binding data. Firstly, the drug dose–receptor occupancy data were plotted and the curves used to derive two doses giving rise to similar degrees of receptor occupancy (which did not differ significantly when compared using an independent samples *t* test). The reduction of ethanol intake by these equivalent occupancy doses was then compared using pairwise contrasts within the ANOVA.

For the DID sucrose consumption data, data were analysed in mls of solution consumed. Acquisition data were analysed using a repeated measures ANOVA across the first four sessions to ensure that the four treatment groups were matched for consumption before treatment. Effect of drug was measured for each drug separately using a repeated measures ANOVA with dose as the within-subject factor.

For the CTA experiments, LiCl data were analysed using a paired *t* test across the two sessions. Data from animals treated with GSK1512498 were analysed with a repeated measures ANOVA with factors for dose (between-subject) and session (within-subject).

## Results

### Home cage alcohol consumption

**Fig. 2:**
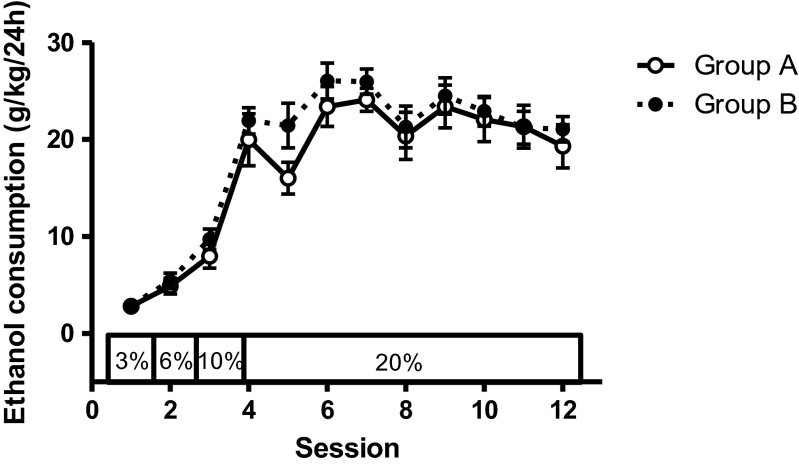
Animals were exposed to ethanol in the home cage using the ethanol escalation procedure. Consumption increased with ethanol concentration and stabilised at the 20 % ethanol solution (average consumption 20.89 ± 2.08 and 21.72 ± 1.35 g/kg in the two groups over the final three sessions). There were no differences between the groups. *Bars* indicate standard errors of the mean (SEM)

Figure 2 shows that daily ethanol intake increased as concentration was increased (significant main effect of session: *F*_11,286_ = 75.58, *p* < 0.001). It is consistent with previous studies that the animals consumed more ethanol over successive sessions. Consumption stabilised at the 20 % concentration, the average ethanol consumption over the final three sessions with 20 % ethanol being 21.05 ± 1.14 g/kg (*n* = 27). When the group status (A or B) coding order of drug administration was included as a covariate, there was no main effect or interaction involving order (main effect of order: *F*_1,25_ = 1.362, *p* = 0.254; order × session *F*_11,275_ = 0.460, *p* = 0.808) showing that the groups were balanced for alcohol consumption before drug testing began.

### Effect of GSK1521498 and naltrexone on ethanol consumption

**Fig. 3:**
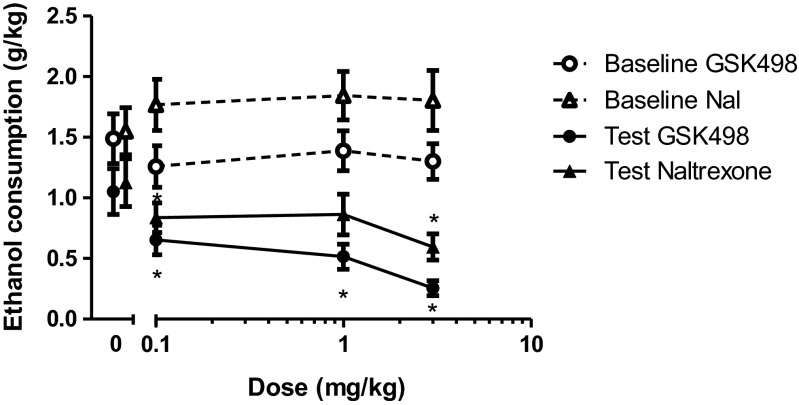
Ethanol consumption following administration of GSK1521498 or naltrexone when the data were combined across phases 1 and 2 of the study: both GSK1521498 and naltrexone produced a significant decrease in ethanol consumption, but this effect was less pronounced following naltrexone administration. Baseline refers to consumption on day prior to respective injection day. Data points for the 0 mg/kg dose have been offset to allow the reader to interpret the error bars.*p < 0.05, significantly different from vehicle. *Bars* indicate SEM. The same data, plotted as percentage change from baseline, are shown in Supplementary Fig. 1

Figure 3 shows average weekly ethanol consumption during DID on both the day prior to drug administration (baseline) and on drug administration days. Table 1 presents the fold changes in ethanol consumption (compared to non-injection days and placebo sessions). The injection procedure produced a small reduction in drinking when compared to non-injection baseline days, but there was no difference among the groups, suggesting that although the injection procedure reduced alcohol consumption, there was no evidence that the order of dosing had effects. There was also a significant difference in consumption between phases (*F*_1,125_ = 34.7, *p* < 0.01), with greater consumption in phase 1. However, there were no significant interactions between phase and other effects of interest, and a sensitivity analysis restricted to only phase 1 data gave similar estimates to those in Table 1, implying that the fold reductions in drinking were consistent irrespective of the baseline drinking level within each phase.

**Table 1:** Fold change in ethanol consumption when animals were administered GSK1521498 or naltrexone when compared with baseline consumption measures

Dose (mg/kg)	Fold decrease in ethanol consumption vs non-injection baseline		Fold decrease in ethanol consumption vs 0 mg/kg dose (baseline-adjusted)	
	GSK1521498	Naltrexone	GSK1521498	Naltrexone
0	1.5 (1.1, 2.1)	1.4 (1.0, 2.0)	–	–
0.1	2.1 (1.5, 2.9)	2.1 (1.5, 3.0)	1.4 (0.9, 2.2)	1.5 (0.9, 2.5)
1	3.3 (2.4, 4.6)	2.5 (1.8, 3.5)	2.2 (1.4, 3.5)	1.8 (1.1, 2.9)
3	5.3 (3.8, 7.4)	2.7 (1.9, 3.9)	3.5 (2.2, 5.6)	1.9 (1.2, 3.1)

Values in brackets are 95 % confidence intervals

Both compounds were associated with a dose-dependent decrease in ethanol consumption. For GSK1521498, post hoc analysis following a significant session by dose interaction (*F*_3,78_ = 6.56, *p* < 0.01) showed that this was due to a dose-dependent effect of drug treatment days only (*F*_3,78_ = 10.106, *p* < 0.001). Each dose of GSK1521498 significantly reduced ethanol intake when compared with vehicle-treated animals (*t*_26_ > 2.76, *p* < 0.01 for all comparisons). In the case of naltrexone, there was also a significant session by dose interaction (*F*_3,78_ = 4.28, *p* < 0.01), which was once again attributable to a dose-dependent effect of drug treatment days only (*F*_3,78_ = 3.56, *p* < 0.05). When compared with vehicle treatment, only the 0.1 and 3 mg/kg dose significantly reduced ethanol consumption (0 vs 0.1 mg/kg, *t*_26_ = 2.23, *p* < 0.05; 0 vs 3 mg/kg, *t*_26_ = 2.96, *p* < 0.01). This decrease in consumption was smaller and not statistically significant when animals were administered the intermediate dose of 1 mg/kg (*t*_26_ = 1.25, *p* > 0.05).

Initial inspection of Fig. 3 and Table 1 suggests apparently different interpretations; the 3 mg/kg dose of naltrexone reduced alcohol intake by 1.2 g/kg, whereas the reduction attributable to GSK1521498 at the same dose is less, approximately 1 g/kg. Nevertheless, when considered as fold changes from baseline, taking fold changes of the simple group means gives 5.2 for GSK1521498 and 3.0 for naltrexone. These values are close to the results of the repeated measures ANOVA in Table 1 (5.3 for GSK1521498 and 2.7 for naltrexone) given that the ANOVA adjusts for other factors such as phase and the within-animal repeated measures.

### Receptor occupancy analysis

**Fig. 4:**
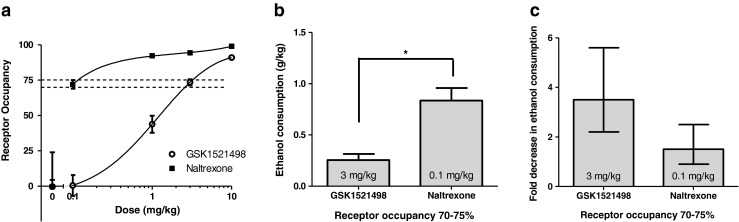
Effects of GSK1521498 and naltrexone on ethanol consumption in the DID procedure when the doses were matched for receptor occupancy. **a** MOPr occupancy in the striatum at different doses of drug. Over the dose ranges used, naltrexone showed greater occupancy than GSK1521498. **b** Ethanol consumption in the DID procedure at doses of drug that produced a 70–75 % occupancy. When the dose of the drug was matched for occupancy, animals treated with GSK1521498 consumed significantly less ethanol than animals treated with naltrexone (*p < 0.001). *Bars* indicate SEM. **c** Fold decrease in ethanol consumption vs 0 mg/kg dose (baseline-adjusted). *Bars* indicate 95 % confidence intervals

In order to compare intrinsic efficacy of the two drugs, receptor occupancy was expressed as a function of drug dose for each drug. Figure 4a shows that naltrexone gave rise to greater receptor occupancies than GSK1521498 over the dose ranges and routes of administration used, suggesting a greater potency. In order to compare the relative efficacies of the two substances, we chose to compare drug effects at doses of each drug giving rise to similar receptor occupancy in the range 70–75 %. For naltrexone, this degree of occupancy corresponded to a dose of 0.1 mg/kg, s.c., whereas an i.p. dose of 3 mg/kg of GSK1521498 gave rise to the same occupancy of striatal receptors (*t*_6_ = 0.34, *p* = 0.749). Figure 4b shows that at this receptor occupancy, GSK1521498 reduced ethanol intake to a greater extent than naltrexone. Figure 4c presents these data as fold change from baseline (*t*_145_ = 3.73, *p* < 0.001).

### Effect of GSK1521498 and naltrexone on sucrose consumption

**Fig. 5:**
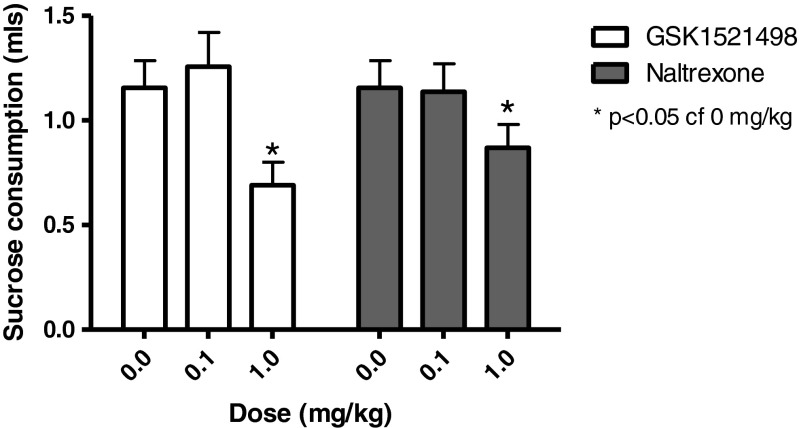
Sucrose consumption following administration of GSK1521498 or naltrexone: there was a significant dose-dependent decrease in sucrose consumption with both GSK1521498 and naltrexone, which became significant at a dose of 1 mg/kg. *p < 0.05

Specificity of the drug effect in reducing ethanol intake was tested using a 2 % sucrose solution in the sipper tubes. During the acquisition phase (first four sessions), animals were not injected. There was a significant increase in sucrose across this period (*F*_3,75_ = 20.01, *p* < 0.001), which stabilised after session 2 (consumption on day 4: 1.6 ± 0.2 ml). Figure 5 shows that both GSK1521498 and naltrexone significantly reduced sucrose consumption at a dose of 1 mg/kg but not 0.1 mg/kg (main effect of GSK1521498: *F*_2,52_ = 8.05, *p* < 0.001; post hoc paired *t* test, 0 vs 1 mg/kg, *t*_26_ = 3.11, *p* < 0.01; main effect of naltrexone: *F*_2,50_ = 3.86, *p* < 0.05; post hoc paired *t* test, 0 vs 1 mg/kg, *t*_25_ = 2.28, *p* < 0.05).

### Conditioned taste aversion

**Fig. 6:**
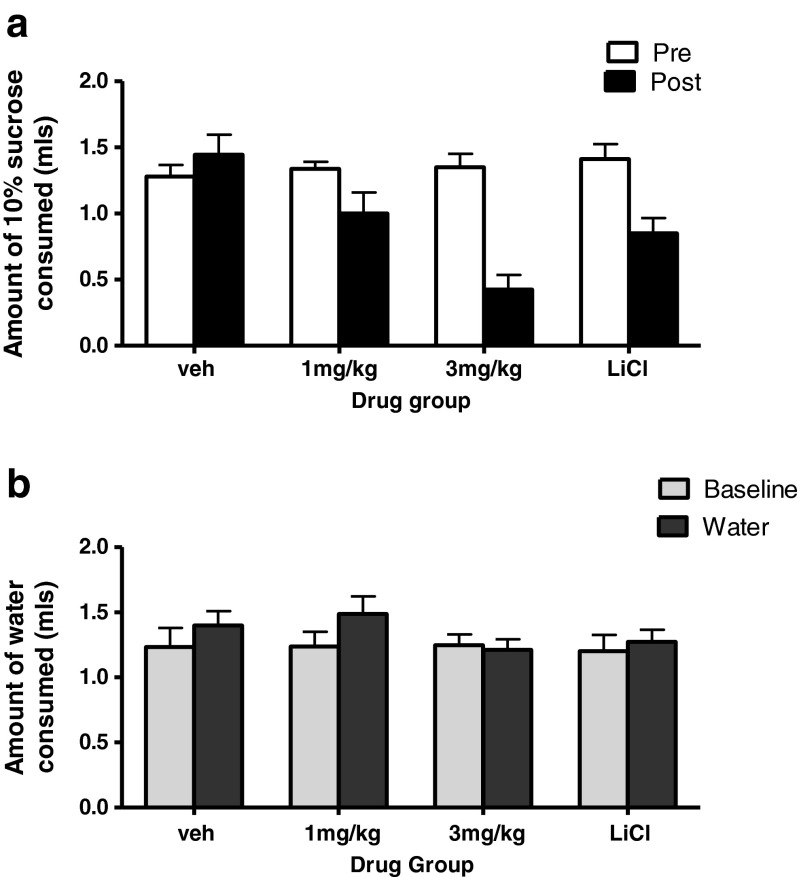
Effect of GSK1521498 and LiCl in supporting a conditioned taste aversion for a sucrose solution in ethanol naive C57BL/6J mice. **a** Sucrose consumed (ml) on the conditioning day (pre) and test day (post). **b** Water consumption (ml) on the specificity test day

A conditioned taste aversion to LiCl was seen as a significant decrease in sucrose consumption on the test day compared with the conditioning day (*t*_7_ = 4.24, *p* < 0.005; Fig. 6).

GSK1521498 produced a conditioned taste aversion only at the highest dose tested (Fig. 6). Repeated measures ANOVA demonstrated a significant main effect of dose (*F*_2,21_ = 6.53, *p* < 0.01), session (*F*_1,21_ = 21.03, *p* < 0.001) and a dose by session interaction (*F*_2,21_ = 15.45, *p* < 0.001). Post hoc analysis showed that GSK1521498 3 mg/kg produced a significant decrease in sucrose consumption on the test day when compared with the conditioning day (*p* < 0.001). A marginal effect was seen with a dose of 1 mg/kg (*p* = 0.06), and no effect was seen after a vehicle injection (*p* = 0.266).

There was no change in water consumption post-conditioning, when compared with baseline drinking levels, in the LiCl-treated animals (*p* = 0.741). Following GSK1521498 treatment, there was a slight increase in water consumption post-conditioning (main effect of session: *F*_1,21_ = 4.97, *p* < 0.05) but this did not reach significance at any single dose.

## Discussion

The mu-opioid receptor system has been shown to play a fundamental role in reward processes. Activation of this system, by both primary and secondary reinforcers, has been implicated in mediating reward and attributing incentive salience to cues associated with delivery of the reinforcer. Therefore, the opioid system has been suggested as a primary target for therapeutic intervention in eating disorders and drug abuse. Indeed, one of the main current pharmacological treatments for alcoholism, naltrexone, has antagonistic effects at MOPr. However, as this treatment has only limited success in treatment of alcohol dependence, further investigation into compounds acting on this target is warranted.

GSK1521498 is currently being developed for disorders of compulsive consumption, including alcohol dependence where patients have a long history of alcohol exposure. Hence, in the current study, we compared the ability of GSK1521498 to that of naltrexone in reducing ethanol consumption in ethanol-experienced animals using a pre-clinical mouse model. Both GSK1521498 and naltrexone reduced ethanol consumption in a dose-dependent manner. A potential weakness of the current study is that we chose to give the two drugs by different routes in order to obtain similar pharmacokinetic profiles, and to align with dosing regimens used in previous studies of GSK1521498 and naltrexone (Ignar et al 2011; Giuliano et al 2012, 2013). This dosing regimen thus allowed comparison of both drugs’ effects on alcohol consumption to their effects at the same doses across a wide range of other animal models of eating behaviour, drug seeking and drug-taking behaviours. However, this approach has the disadvantage that direct comparison of relative potencies of the two drugs is not possible. We sought to overcome this potential weakness by determining ex vivo receptor occupancies across the dose range chosen, allowing us to compare the pharmacodynamic effects at similar occupancy levels, thus controlling for potential differences between the two drugs in absorption, metabolism or blood-brain barrier penetration. At doses at which both drugs achieved approximately 70–75 % receptor occupancy, the reduction in alcohol consumption was significantly greater in animals treated with GSK1521498 compared with naltrexone (2.5-fold difference).

Analysis comparing drug action at the same receptor occupancy level is seldom performed and yet offers a potential in vivo measure of drug relative efficacy. This is especially important when drugs are administered by different routes and with different pre-treatment times as in the present experiment. Nevertheless, only one pair of doses (0.1 mg/kg sc naltrexone and 3 mg/kg i.p. GSK1521498) was approximately matched for 70–75 % receptor occupancy, and in future studies, it would be valuable to plan dosing a priori to match for receptor occupancy over a wider range of occupancy levels so that differential intrinsic efficacy can be assessed at more clinically relevant doses achieving more complete blockade of the mu-opioid receptor.

This ability to achieve a greater effect at equivalent occupancies suggests that GSK1521498 may possess a higher intrinsic efficacy than naltrexone in opposing MOPr function. Higher intrinsic efficacy might come about if naltrexone possesses partial agonist properties at the MOPr (Kelly et al. 2014), and GSK1521498 is either a neutral antagonist (Kelly et al. 2014) or possesses partial inverse agonism at MOPR1 (Ignar et al. 2011). Although Kelly et al. (2014) failed to find evidence for inverse agonist activity at endogenous MOPr in brain tissue from drug-naive mice (Kelly et al. 2014), GSK1521498 did demonstrate mild inverse agonist activity in brain tissue from mice that had received morphine pre-treatment. Under the same experimental conditions, naltrexone did not behave as an inverse agonist (Kelly et al. 2014). It might be worth noting that the mice in our study had been exposed to considerable ethanol intake prior to the pharmacological experiments, but we are unaware of data to suggest that such ethanol exposure mimics morphine exposure in increasing constitutive activity at MOPrs, thus allowing inverse agonism to be revealed.

A complexity arises in the interpretation of the key experiment in this study in that the injection procedure produced a reduction in drinking when compared to non-injection baseline days, even when animals were dosed with vehicle and no active drug. Although the reduction is relatively small and similar for both vehicles studied, there is some variation in the consumption on non-injection days at different doses which could not be ignored in the analysis since failing to adjust for non-injection consumption would lead to overestimating the effect of GSK1521498 relative to naltrexone. It was also observed that when consumption was low, the within- and between-animal variability was also low, and vice versa at high consumptions. These factors together necessitated a log-transformation of consumption data prior to ANOVA and reporting results as fold changes adjusting for both non-injection consumption and vehicle consumption.

The results in the present study show a slight decrease in the effectiveness of naltrexone when compared with other studies in the literature. Indeed, Kamdar et al. (2007) reported that naltrexone, 1 mg/kg, decreased consumption of 20 % ethanol over a 2-h period using the DID protocol. The decreased potency in the present study may be due to the repeated administration of naltrexone, which has been shown to lead to a reduction in its ability to reduce ethanol intake (Middaugh and Bandy 2000). Indeed, Phillips et al. (1997) reported an increase in ethanol intake following chronic treatment with naltrexone in C57BL/6J mice.

It should also be noted that both GSK1521498 and naltrexone, at a dose of 1 mg/kg, significantly reduced sucrose consumption, although the magnitude of reduction was similar for both drugs. This result was not surprising as GSK1521498 has been shown to decrease both nocturnal food consumption and preference for sucrose containing solutions in animal studies (Giuliano et al. 2012; Ignar et al. 2011), and to decrease the hedonic response to sweetened dairy products in humans (Ziauddeen et al. 2013). Naltrexone has also been shown to decrease food intake (for review, see Berner et al. 2011). However, in the study by Kamdar and colleagues (2007), where a similar DID protocol was used, naltrexone did not reduce consumption of either 10 % sucrose or plain water. Although no data currently exist in this field, it would be interesting to see if repeated treatment with naltrexone enhances its effect on sucrose consumption whilst diminishing its effect on ethanol consumption. Although there was evidence for higher efficacy of GSK1521498 versus naltrexone on alcohol consumption, there was no significant difference in the effects of the two drugs on sucrose consumption. A previous study found that both drugs had effects on food seeking and binge-like eating behaviours, and GSK1521498 specifically had effects on incentive motivation for chocolate (Giuliano et al. 2012). Further studies will be needed to clarify whether GSK1521498 differs significantly from naltrexone in its effects on sweet and fat food seeking and consumption.

Besides these data on alcohol and sucrose consumption, the current study does not provide new results from models of other consummatory behaviours; but the same drugs have been studied at the same doses in several prior studies of eating behaviour, chocolate-, cocaine- and heroin-seeking behaviours, and cocaine and heroin self-administration (Ignar et al 2011; Giuliano et al 2012, 2013). Consistently, GSK1521498 has been shown to attenuate behavioural measures of drug or food reward-seeking and to reduce consumption of food and heroin. In clinical studies of healthy volunteers and binge-eating obese patients (Nathan et al. 2012a, b). GSK1521498 has also been shown to reduce the hedonic and consummatory response to food (especially high fat foods) and to reduce brain functional activation measured by fMRI following oral administration of a small quantity of (rewarding) fruit juice during scanning (Rabiner et al. 2011). On this basis, we suggest that GSK1521498 does not selectively modulate alcohol-related behaviours but has more general effects on opioid-mediated reward signalling in the brain that are behaviourally manifest by reduced seeking and consuming of many rewarding substances.

Decreases in consumption of ethanol (or other rewards) could be due to non-specific effects, such as general malaise following the injection, and there is some evidence to suggest that naltrexone may support conditioned taste aversion (CTA) at relatively low doses (Parker and Rennie 1992). Consistent with that notion, administration of GSK1521498 following exposure to a novel sucrose solution, reduced consumption of that solution, but not of water, on a subsequent occasion. This is consistent with GSK1521498 possessing aversive properties that become conditioned to the taste of sucrose. Nevertheless, the ability of GSK1521498 to support a conditioned taste aversion was seen only at a high dose of 3 mg/kg (at which it was more effective than a standard dose of LiCl), whereas its ability to reduce ethanol consumption was already apparent at a dose of 0.1 mg/kg (Fig. 3). It thus seems unlikely that GSK1521498’s effects on alcohol consumption simply reflect induction of malaise.

In summary, the current study showed that both GSK1521498 and naltrexone reduce ethanol consumption in an experimental model of drinking to excess, with evidence for greater intrinsic efficacy of GSK1521498.

## Electronic supplementary material

Supplementary Fig. 1 (DOCX 49 kb)
