# Supplementary material for: The novel mu-opioid antagonist, GSK1521498, reduces ethanol consumption in C57BL/6J mice
Source: Psychopharmacology (Berl). 2015 Jul 5;232(18):3431–41. doi: 10.1007/s00213-015-3995-x (PMC4537503; doi:10.1007/s00213-015-3995-x)
Supplement: Supplementary file 1 — (DOCX 49 kb) [file 213_2015_3995_MOESM1_ESM.docx]

Supplementary Figure 1

Data from Figure 3, replotted to show effects of treatment in reducing ethanol consumption relative to the test baseline. Bars represent SEM.
